# Gram-Scale Synthesis of Blue-Emitting CH_3_NH_3_PbBr_3_ Quantum Dots Through Phase Transfer Strategy

**DOI:** 10.3389/fchem.2018.00444

**Published:** 2018-09-26

**Authors:** Feng Zhang, Changtao Xiao, Yunfei Li, Xin Zhang, Jialun Tang, Shuai Chang, Qibing Pei, Haizheng Zhong

**Affiliations:** ^1^Beijing Key Laboratory of Nanophotonics and Ultrafine Optoelectronic Systems, School of Materials Science and Engineering, Beijing Institute of Technology, Beijing, China; ^2^Department of Materials Sciences and Engineering, California NanoSystems Institute, Henry Samuli School of Engineering and Applied Science, University of California, Los Angeles, Los Angeles, CA, United States

**Keywords:** emulsion synthesis, CH_3_NH_3_PbBr_3_, quantum dots, blue-emitting, phase transfer

## Abstract

Reprecipitation synthesis has been demonstrated to be a simple and convenient route to fabricate high quality perovskite quantum dots toward display applications, whereas the limited chemical yields (< 10%) and difficulty of purification limited its further application. In order to overcome this issue, we here report a modified emulsion synthesis by introducing phase transfer strategy, which achieving effective extraction of newly formed perovskite quantum dots into non-polar solvent and avoiding the degradation of perovskite quantum dots to a large extent. Based on this strategy, gram-scale CH_3_NH_3_PbBr_3_ quantum dots were fabricated in 10 mL (~0.02 mol/L) colloidal solution with chemical yields larger than 70%. The as fabricated CH_3_NH_3_PbBr_3_ quantum dots exhibit an emission peak of 453 nm and a full width at half maximum of only 14 nm. Moreover, electroluminescent devices based on blue emitting CH_3_NH_3_PbBr_3_ quantum dots were also explored with a maximum luminance of 32 cd/m^2^, showing potential applications in blue light emitting devices.

## Introduction

The development of lighting and display technologies demand luminescent materials with high color quality (Lin and Liu, 2011; Shirasaki et al., 2013; Pust et al., 2014). In the past 3 years, halide perovskite quantum dots (QDs) have emerged as a new generation of luminescent materials with excellent photoluminescent (PL) properties such as high quantum yields (QYs), panchromatic wavelength tunability and narrow emission line width (Protesescu et al., 2015; Stranks and Snaith, 2015; Zhang et al., 2015), which make them promising candidates for wide color gamut displays (Bai and Zhong, 2015; Kim et al., 2016; Li et al., 2017). To further promote their potential commercialization applications, efficient mass production of colloidal perovskite QDs has become an important research topic (Huang H. et al., 2016; Xing et al., 2016; Ha et al., 2017; Zhang et al., 2017a). Great progress has been made on the colloidal synthesis of perovskite QDs (Huang et al., 2015a; Leng et al., 2016; Wang et al., 2016; Polavarapu et al., 2017; Protesescu et al., 2017) where two main synthesis strategies have been established, namely, high temperature hot-injection and room temperature reprecipitation. Currently room temperature reprecipitation methods including ligand-assisted reprecipitation (LARP) (Zhang et al., 2015) and emulsion reprecipitation (Huang et al., 2015a) have been more universally applied owing to their simple and low temperature synthesis process and feasibility for both organic-inorganic hybrid and all-inorganic perovskite materials (Lignos et al., 2016; Wei et al., 2016; Levchuk et al., 2017a,b; Minh et al., 2017). However, there still exist many challenges that hinder the development of perovskite QDs for display applications. In 2015, our group fabricated CH_3_NH_3_PbBr_3_ QDs by applying the conventional emulsion strategy (Huang et al., 2015a). However, the obtained CH_3_NH_3_PbBr_3_ QDs through this strategy were precipitated in the mixture of polar and non-polar solvents. It is widely recognized that perovskite QDs can be destroyed by even trace amount of polar solvent, which leads to limited chemical yields (Huang H. et al., 2016; Zhang et al., 2017a). On the other hand, the conventional emulsion process normally generate multiple products from nano-sized QDs to micro-sized crystals, thus the purification process by centrifuging will inevitably lead to material loss. Therefore, the extraction of the perovskite QDs from polar solvent as well as the reduction of side products is a crucial step to enhance the chemical yield. From the perspective of electroluminescent (EL) device integration, the reported green emission EL device based on halide perovskite QDs or nanocrystals has achieved an EQE over 16%, (Han et al., 2018; Yang et al., 2018) however, there still lacks high quality blue emission perovskite materials, especially the ones with controlled emission wavelength within the pure blue range of 450–470 nm. On the other hand, the reported blue EL emission perovskite materials so far are all based on 2D or quasi 2D structure with the assistance of large organic ammonium molecules, (Kumar et al., 2016; Wang et al., 2017) no blue EL result based on pure 3D structured CH_3_NH_3_PbBr_3_ QDs was reported. Therefore, further exploration of colloidal chemistry to overcome these issues is imperative.

Here, for the first time we report the gram scale fabrication of 3D structured blue emitting CH_3_NH_3_PbBr_3_ QDs through a modification of the conventional emulsion synthesis method. The main alteration is that acetonitrile (ACN) is used as demulsifier instead of acetone. As ACN is miscible with N, N-dimethylformamide (DMF) but immiscible with hexane, the addition of ACN initiates the crystallization for CH_3_NH_3_PbBr_3_ QDs and also induces phase separation at the same time. As a result, the as-formed CH_3_NH_3_PbBr_3_ QDs in DMF and ACN phase spontaneously transferred into hexane phase, leading to enhanced chemical yields and simplified purification process by stratification. Based on the modified emulsion route, strong blue-emitting CH_3_NH_3_PbBr_3_ QDs with an average diameter of 2.4 nm was successfully fabricated. The obtained CH_3_NH_3_PbBr_3_ QDs exhibit an emission peak of 454 nm and full width at half maximum (FWHM) of only 14 nm. Furthermore, LED devices based on the blue perovskite QDs were explored, demonstrating their potential in display applications.

## Materials and methods

### Chemicals

PbBr_2_ [lead(II) bromide, 99%, alfa aesar],methylamine (CH_3_NH_2_, 33 wt. % in absolute ethanol, aladdin), n-octylamine (≥99%, aladdin), hydrobromic acid(HBr, 49 wt.% in water, alfa aesar),oleic acid (≥90%, Alfa aser), N, N-dimethylformamide (DMF, analytical grade, Beijing Chemical Reagent Co., Ltd., China), acetronitrile (ACN, analytical grade, Beijing Chemical Reagent Co., Ltd., China), toluene (analytical grade, Beijing Chemical Reagent Co., Ltd., China).

### Fabrication of hybrid CH_3_NH_3_PbBr_3_ QDs

CH_3_NH_3_Br was synthesized according to the literature (Zhang et al., 2015). Colloidal CH_3_NH_3_PbBr_3_ QDs was fabricated by a modification of the reported emulsion synthesis. Firstly, PbBr_2_ (0.2 mmol, 0.0734 g) and CH_3_NH_3_Br (0.2 mmol, 0.0224 g) were dissolved in 0.5 mL DMF and sonicated for 10 min to form solution A. Solution B was prepared by mixing of n-hexane (10 mL), n-octylamine (30 μL) and oleic acid (35 μL). Then, the solution A was dropwise added into solution B under vigorous stirring. With the addition of solution A, the color of mixed solution gradually turned from clear to slight milky indicating the formation of emulsion system. Then, 6 mL of ACN was added into the emulsion system as demulsifier to initiate demulsion process. The limited solubility of CH_3_NH_3_PbBr_3_ precursors in ACN drives the formation of CH_3_NH_3_PbBr_3_ QDs. The mixture stratified into two phases after removing the stir. The top layer was collected as dispersion of CH_3_NH_3_PbBr_3_ QDs in hexane for further characterization. Furthermore, CH_3_NH_3_PbBr_3_ QD powder can be obtained by collecting the precipitates after adding a fixed amount of ethyl acetate into the QDs dispersion in hexane solution.

### Fabrication and characterization of EL devices

ITO substrate was sequentially washed with acetone, ethanol and deionized water, followed by plasma treatment for 5 min. Poly(3,4-ethylenedioxythiophene):poly(styrenesulfonate) (PEDOT:PSS) solution was spin coated on the ITO film at 4000 rpm, then annealed at 150°C for 15 min. CH_3_NH_3_PbBr_3_ QDs dispersed in hexane (2.5 mg/mL) was spin-coated on the PVK film, followed by thermal annealing at 70°C for 15 min, then (2,2,2-(1,3,5-benzinetrily)tris(1-phenyl-1-Hbenzimidazole) (TPBi) (40 nm), CsF (1 nm) and Al (80 nm) were thermally deposited in sequence in a high-vacuum chamber with a deposition rate of 1, 0.1, and 5 Å/s, respectively (< 10^−6^ mbar). Keithley 2400 and Keithley 2000 SourceMeter unit linked to a calibrated silicon photodiode were used to measure the current-voltage-brightness characteristics. A spectrophotometer PR-655 (Photo Research, Inc.) was used to measure the electroluminescence spectrum.

### Characterizations

UV-Vis absorption spectra were measured on a UV-6100 UV-Vis spectrophotometer (Shanghai Mapada Instruments Co.,Ltd., China). X-ray diffraction patterns (XRD) were measured on a Bruker/D8 FOCUS X-ray diffractometer with Cu Kα radiation source (wavelength at 1.5406 Å). The samples were scanned from 3° < 2θ < 60° at an increment of 2°/min. Liquid samples of toluene solutions deposited on amorphous carbon-coated copper grids were analyzed using a JEOL-JEM 2100F transmission electron microscopy (TEM)operating at an acceleration voltage of 200 kV. PL spectra were taken using a F-380 fluorescence spectrometer (Tianjin Gangdong Sci. & Tech. Development. Co., Ltd., China). Time-resolved PL was collected using fluorescence lifetime measurement system (C11367-11, Hamamatsu Photonics, Japan) with excitation wavelength of 405 nm. The absolute PLQYs of diluted QDs solutions were determined using a fluorescence spectrometer with integrated sphere (C9920-02, Hamamatsu Photonics, Japan) under blue light emitting diodes (LED) excited at a wavelength of 450 nm.

## Results and discussion

### Modified emulsion synthesis process with phase transfer strategy

Emulsion system, first proposed by Schulman and co-workers in 1959, consists of at least three components namely polar solvent, non-polar solvent and surfactant (Schulman et al., 1959). After developing for over 50 years, emulsion strategy has become one of the versatile preparation techniques for the synthesis of numerous organic and inorganic nanoparticles (Malik et al., 2012). The physical model of an emulsion is that the surfactant forms an interfacial layer between the polar and the non-polar solvents and let the polar solvent dispersed in non-polar solvent as small droplets at microscopic level. These micrometer-sized small droplets can act as small nanoreactors and control the size, morphology, and surface area of nanoparticles (Lopez-Perez et al., 1997).

The fabrication of blue emitting CH_3_NH_3_PbBr_3_ QDs was based on the modification of our reported emulsion synthesis (Huang et al., 2015a). In a typical emulsion synthesis process, DMF and hexane are selected as good and poor solvent for CH_3_NH_3_PbBr_3_ precursors. Oleic acid and octylamine act as surfactants and *t*-butanol or acetone is used as demulsifier. Owing to the immiscibility between DMF and hexane, CH_3_NH_3_PbBr_3_ precursors in DMF can be dispersed as microscopic droplets in hexane under vigorous stirring. The subsequent addition of demulsifier (acetone) provides the driving force for crystallization of CH_3_NH_3_PbBr_3_ QDs. However, the chemical yields of CH_3_NH_3_PbBr_3_ QDs through this emulsion system is lower than 10%. In fact, the conventional emulsion synthesis method also produces micrometer-sized and even larger CH_3_NH_3_PbBr_3_ particles which occupy a large proportion of the overall products, as shown in Figure S1. This is because that the addition of acetone induces instant solvent mixing process, leading to a large degree of supersaturation for CH_3_NH_3_PbBr_3_ precursors. From the viewpoints of classical nucleation and growth theory, the high supersaturation degree will bring fast nucleation and growth rates for CH_3_NH_3_PbBr_3_ precursors, which lacks feasibility to control the crystallization process (Barlow et al., 2004; Leite and Ribeiro, 2011; Thanh et al., 2014; Zhang et al., 2017b). On the other hand, the residual solvents (DMF and acetone) after solvent mixing can degrade the as-formed CH_3_NH_3_PbBr_3_ QDs, (Huang S. et al., 2016; Vybornyi et al., 2016; Chiba et al., 2017) resulting in even lower chemical yields.

**Figure 1:**
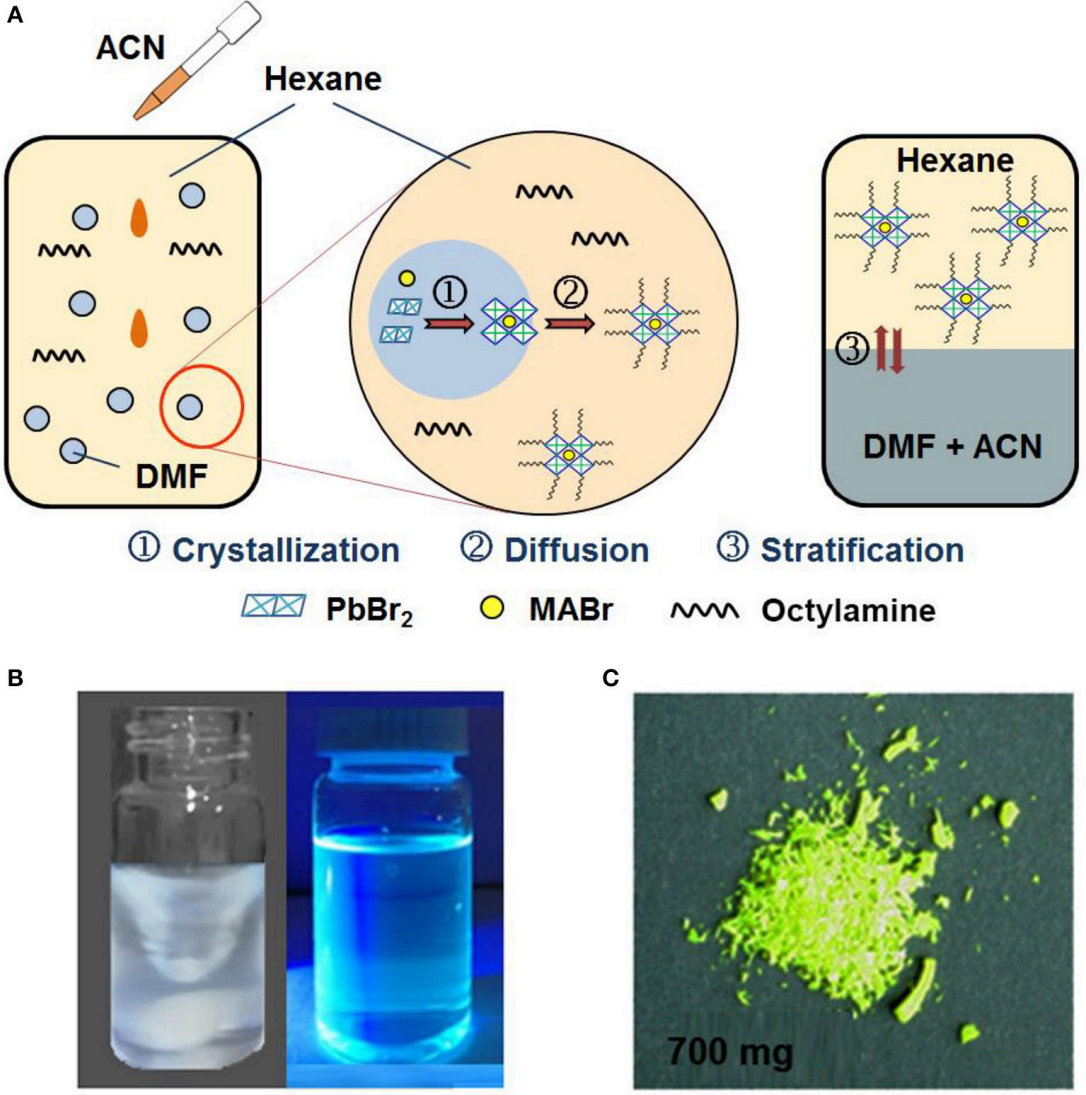
**(A)** Schematically illustration of the modified emulsion synthesis process with phase transfer strategy. **(B)** Optical photographs of the established emulsion system after addition of ACN (left) and obtained CH_3_NH_3_PbBr_3_ QDs dissolved in hexane under UV 365 nm radiation. **(C)** Optical photograph of resultant CH_3_NH_3_PbBr_3_ QDs powder in one pot synthesis.

It can be suggested that if the newly formed CH_3_NH_3_PbBr_3_ QDs can be separated from their nucleation area and promptly transferred into another phase, the degradation of CH_3_NH_3_PbBr_3_ QDs by contacting good solvent can be avoided. Here, we replaced the demulsifier acetone with ACN and modified the emulsion synthesis through a phase transfer strategy accordingly. Figure 1 schematically illustrate the synthetic process of CH_3_NH_3_PbBr_3_ QDs by emulsion synthesis with phase transfer strategy. Typically, ACN instead of acetone is added into the emulsion system to induce the demulsion process. As ACN is miscible with DMF but immiscible with hexane, the added ACN mixes with the DMF droplet and drives the nucleation and growth process of CH_3_NH_3_PbBr_3_ QDs due to the limited solubility of CH_3_NH_3_PbBr_3_ precursors in ACN. The formed CH_3_NH_3_PbBr_3_ QDs spontaneously diffuses into hexane solvent phase. Such phase separation process greatly impede the further growth and aggregation of the QDs into large particles, which is the primary cause of the enhanced chemical yields. Finally, after removed from magnetic stirrer, the mixed solution can be stratified into two layers. As shown in the right most part of Figure 1A, the bottom layer is the mixture of DMF and ACN due to their relatively larger density, while the top layer is CH_3_NH_3_PbBr_3_ QDs/hexane dispersion. Powdery CH_3_NH_3_PbBr_3_ QDs can be easily obtained by adding ethyl acetate as precipitator without the further purification process as in the conventional emulsion synthesis (Protesescu et al., 2017). In a word, precise control for CH_3_NH_3_PbBr_3_ QDs is achieved through this modified emulsion synthesis with phase transfer strategy and the chemical yield is calculated to be more than 70%, producing ~700 mg CH_3_NH_3_PbBr_3_ QDs powder per batch in 10 mL (~0.02 mol/L) colloidal solution (the weight of the starting material in the solvent is about 1,000 mg). Moreover, this modified emulsion synthesis method can also be applied for the fabrication of other types of perovskite QDs. As shown in Figure S2, high quality CH(NH_2_)_2_PbBr_3_ (FAPbBr_3_) QDs were also fabricated, indicating the wide applicability of this method.

### Structure and optical properties of as-prepared CH_3_NH_3_PbBr_3_ QDs

**Figure 2:**
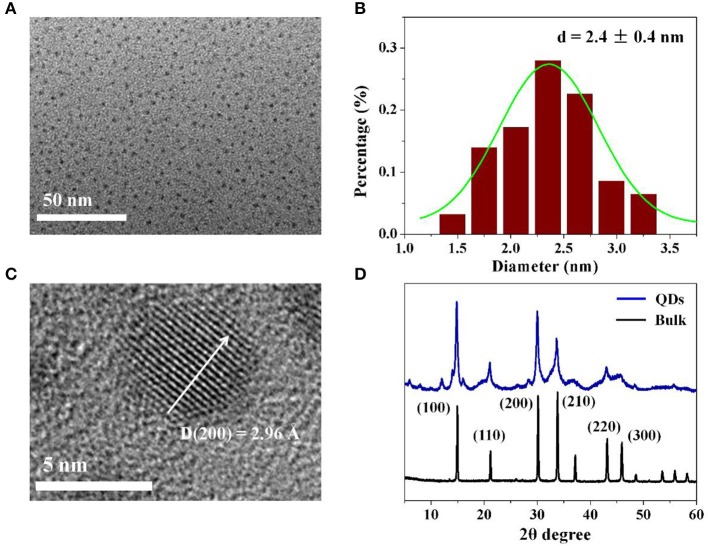
**(A)** TEM image of as-synthesized colloidal CH_3_NH_3_PbBr_3_ QDs. **(B)** Statistical analysis of **(A)**. **(C)** High resolution TEM image of single CH_3_NH_3_PbBr_3_ QD. **(D)** XRD spectra of colloidal CH_3_NH_3_PbBr_3_ QDs and the corresponding CH_3_NH_3_PbBr_3_ bulk.

The as fabricated CH_3_NH_3_PbBr_3_ QDs were further characterized by applying TEM and XRD measurements. From the TEM image in Figure 2A and statistical analysis in Figure 2B, it is observed that the obtained CH_3_NH_3_PbBr_3_ QDs have an average diameter of 2.4 nm with size deviation of ± 0.4 nm. The high resolution TEM image of a CH_3_NH_3_PbBr_3_ QD in Figure 2C shows a interplanar distance of 2.96 Å, which is consistent with the (200) crystal face. In Figure 2D, CH_3_NH_3_PbBr_3_ QDs show main diffraction peaks of 15.04, 21.29, 30.26, 33.92, 37.25, 43.30, and 45.98°, which can be well matched with corresponding bulk CH_3_NH_3_PbBr_3_ in cubic phase (Pm3m) (Stoumpos et al., 2013). The XRD and TEM results demonstrate the formation of highly crystalline CH_3_NH_3_PbBr_3_ QDs.

**Figure 3:**
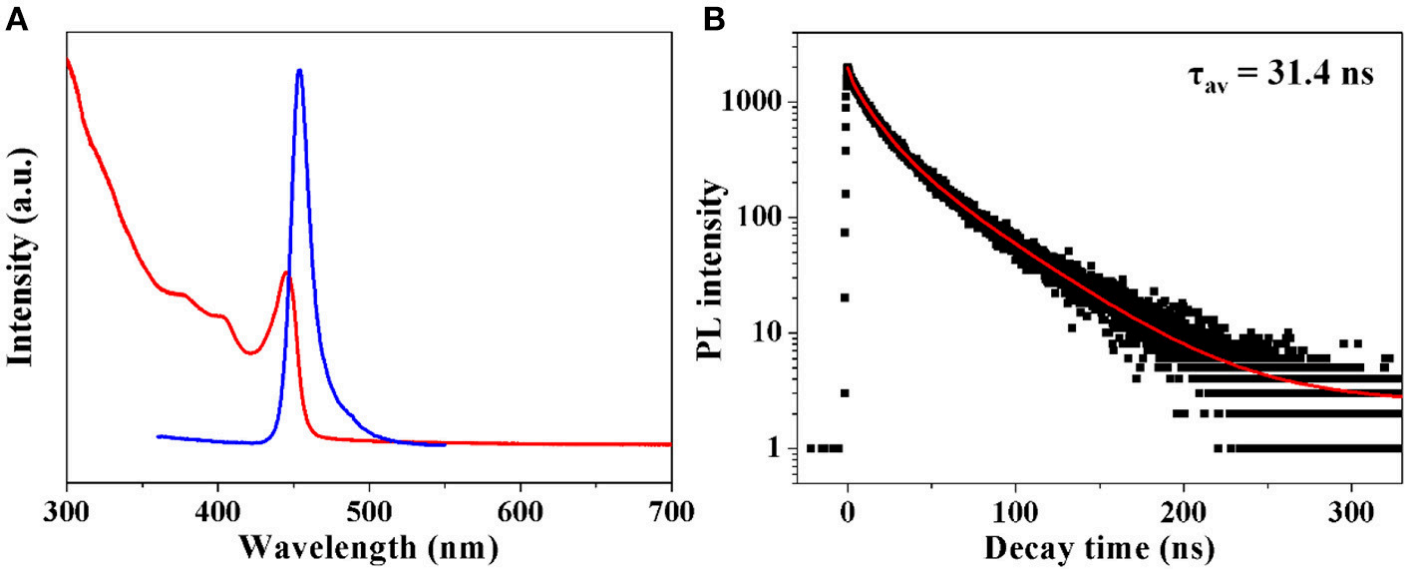
**(A)** Absorption and PL spectra of as fabricated CH_3_NH_3_PbBr_3_ QDs. **(B)** Time-resolved PL spectrum of CH_3_NH_3_PbBr_3_ QDs.

UV-vis absorption, steady state and time-resolved PL are employed to investigate the optical properties of the as fabricated CH_3_NH_3_PbBr_3_ QDs. From Figure 3A, it is observed that colloidal CH_3_NH_3_PbBr_3_ QDs show a typical excitonic peak at 440 nm. An emission peak of 454 nm with a FWHM of 14 nm can also be identified. The absolute PLQYs of these obtained CH_3_NH_3_PbBr_3_ QDs were determined to be ~15%, which were comparable to the reported lead free (CH_3_NH_3_)_3_Bi_2_Br_9_ QDs (Leng et al., 2016). Moreover, the emission wavelength can be tuned by varying the amount of ligands. As shown in Figure S3, the emission peak decreased from 510 to 430 nm with the amount of ligands varied from 10 to 100 μL. The blue-shift of emission peaks can be well explained by the quantum size effect in CH_3_NH_3_PbBr_3_ QDs (Di et al., 2015; Huang et al., 2015b; Malgras et al., 2016). It has been demonstrated that CH_3_NH_3_PbBr_3_ QDs with size close to 2 nm will exhibit strong quantum confinement effect (Tanaka et al., 2003; Sichert et al., 2015). The average diameters of literature reported green emissive CH_3_NH_3_PbBr_3_ QDs are mostly larger than 3 nm. As shown in Figure S4, the blue emitting CH_3_NH_3_PbBr_3_ QDs have relatively smaller sizes. The confirmed average particle size of 2.4 nm locates in strong quantum confinement range, which contributes to the blue shifted emission. To further acquire information on carrier recombination dynamics, time-resolved PL was conducted. The decay curve in Figure 3B was fitted by using multi-exponential functions (Equation 1) and the average lifetime (τ_av_) was determined by Equation 2.

(1) $$\text{I}=\sum_{\text{i}}{\text{A}}_{\text{i}}\exp\left(\text{-t/}\tau_{\text{i}}\right)\;\text{i}=1,\text{2},\text{3}\;\ldots$$

(2) $$\begin{array}{r} {\text{τ}}_{\text{av}}=\underset{\text{i}}{\sum }\frac{A_{i}\tau_{i}^{2}}{A_{i}\tau_{i}}i=1,2,3\;\ldots \end{array}$$

Halide perovskites have been known to show tunable features from exciton to free carries (D'Innocenzo et al., 2014a; Stranks et al., 2014). It has been demonstrated that the free carrier recombination often gives an average lifetime from hundreds to thousands of nanoseconds while the exciton recombination exhibits an average lifetime of tens of nanoseconds (D'Innocenzo et al., 2014a,b; Stranks et al., 2014). The detected average lifetime of 31.4 ns from the fitting result implies that the exciton recombination would occupy a large fraction in as fabricated CH_3_NH_3_PbBr_3_ QDs, which is consistent with their relatively high PL QYs.

### Performance of CH_3_NH_3_PbBr_3_ QDs based EL devices

**Figure 4:**
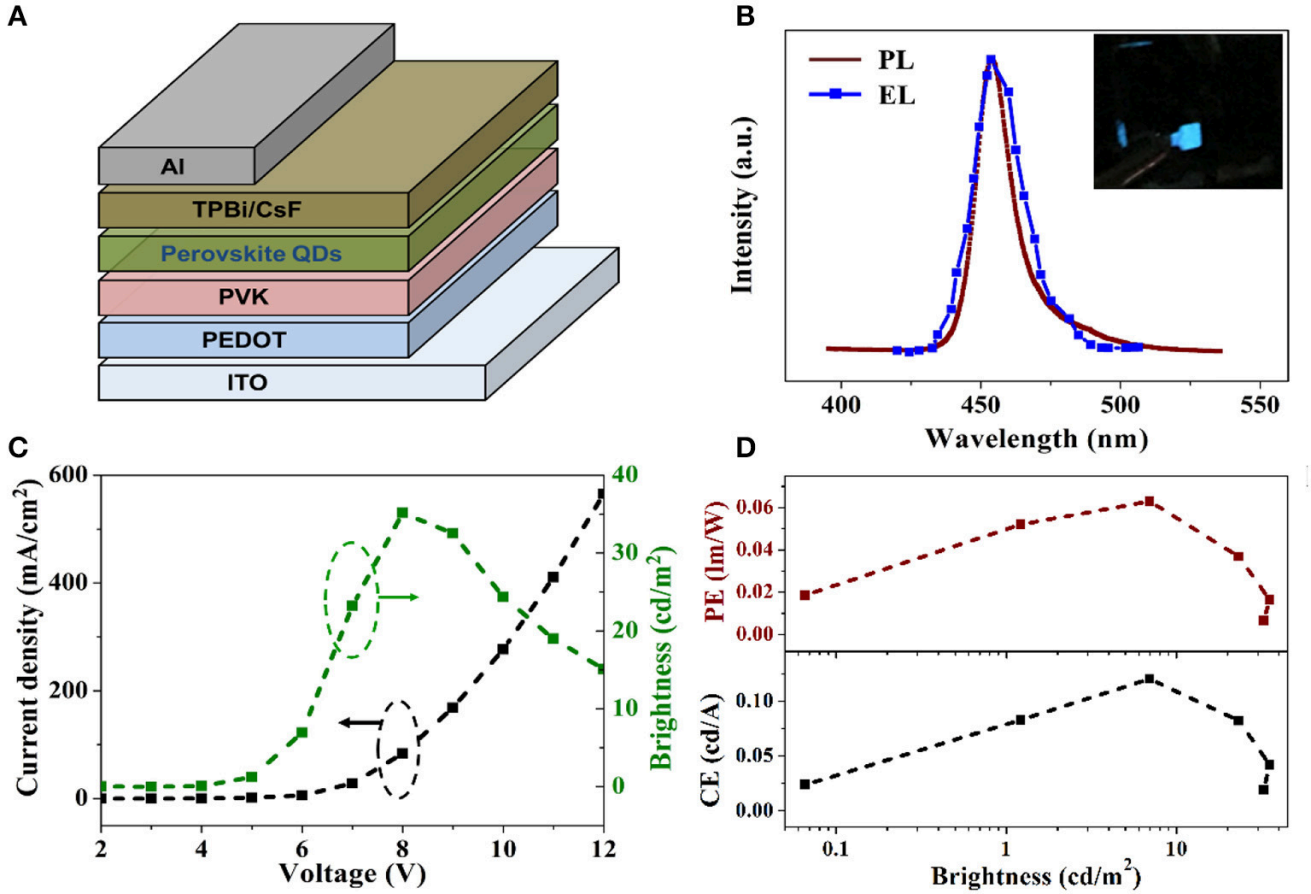
**(A)** Schematically illustration of the device structure employed in the QD-LED. **(B)** Comparison of normalized EL (solid red line) and PL (dashed blue line) spectra of CH_3_NH_3_PbBr_3_ QDs. The inset is an optical photograph of a lit QD-LED. **(C)** Current density (black) and brightness (green) vs. voltage. **(D)** Current, power efficiency, and EQE as a function of brightness of the QD-LED.

EL devices based on blue emitting CH_3_NH_3_PbBr_3_ QDs were also fabricated by applying a simple sandwich structure of ITO/PEDOT/PVK/CH_3_NH_3_PbBr_3_ QDs/TPBi/CsF/Al, as shown in Figure 4A. Poly(3,4-ethylenedioxythiophene):polystyrenesulfonate (PEDOT:PSS) was used to enhance the hole injection. 1,3,5-tris (2-*N*-phenylbenzimidazolyl) benzene (TPBi) was used as electron transporting layer (ETL) with CsF as cathode buff layer. CH_3_NH_3_PbBr_3_ QDs dissolved in hexane was deposited on the top of PVK layer by spin-coating. Figure 4B shows the comparison between PL spectrum of CH_3_NH_3_PbBr_3_ QDs in hexane and EL spectrum of resulting LED devices. Compared with the previously reported deep blue (<440 nm) or sky blue (>480 nm) perovskite EL devices, the 454 nm EL peak of our device is located in the pure blue range and thus more suitable for display integration. It is also noted that the EL spectrum exhibits the same emission peak with the PL spectrum, indicating that the EL is totally originated from the exciton recombination in CH_3_NH_3_PbBr_3_ QDs film during device operation. The FWHM of the EL spectrum is slightly broadened to 16 nm, which is a common phenomena in LED devices (Lee et al., 2014; Zhang et al., 2016). Figure 4C presents the voltage-dependent curves of current density and luminance for CH_3_NH_3_PbBr_3_ QDs based LEDs. The turn on voltage is identified to be 4 V. The current densities varies from 0 to 37 mA/cm^2^ with voltage increased from 2 to 12 V. The as fabricated blue emitting LEDs reached its maximum brightness of 32 cd/m^2^ at 8 V. The brightness dependent current efficiency and power efficiency were plotted in Figure 4D. The maximum current efficiency and maximum power efficiency are calculated as 0.12 cd/A and 0.063 lm/W, respectively. Further work is underway to improve the efficiencies, brightness and stability of CH_3_NH_3_PbBr_3_ QDs based LEDs.

## Conclusions

In summary, CH_3_NH_3_PbBr_3_ QDs with average size of 2.4 nm were successfully fabricated via a modification of conventional emulsion synthesis process. Through introduction of phase transfer strategy, the chemical yields of the final product was greatly improved to larger than 70%, which contributes to achieve gram scale synthesis in a 10 mL emulsion system (0.02 mol/L). The obtained CH_3_NH_3_PbBr_3_ QDs exhibit strong blue emission peaked at 454 nm with a FWHM of 14 nm. Moreover, EL devices based on blue emitting CH_3_NH_3_PbBr_3_ QDs were also fabricated with a maximum luminance of 32 cd/m^2^, demonstrating their potential applications as alternative blue-emitting materials for display applications.

## Author contributions

FZ, CX, and XZ designed the experiments. FZ, SC, JT, and YL analyzed the results. FZ, SC, QP, and HZ wrote and revised the manuscript. All authors have approved the final revised manuscript.

### Conflict of interest statement

The authors declare that the research was conducted in the absence of any commercial or financial relationships that could be construed as a potential conflict of interest.
