# Supplementary material for: Gram-Scale Synthesis of Blue-Emitting CH3NH3PbBr3 Quantum Dots Through Phase Transfer Strategy
Source: Front Chem. 2018 Sep 26;6:444. doi: 10.3389/fchem.2018.00444 (PMC6169035; doi:10.3389/fchem.2018.00444)
Supplement: Supplementary file 1 [file Table_1.docx]

**Supporting Information**

Gram-Scale Synthesis of Blue-Emitting CH_3_NH_3_PbBr_3_ Quantum Dots through Phase Transfer Strategy

*Feng Zhang,*[*^†^*](http://pubs.acs.org/doi/abs/10.1021/jp072540p?prevSearch=%2528Gregory%2BScholes%2529%2BNOT%2B%255Batype%253A%2Bad%255D%2BNOT%2B%255Batype%253A%2Bacs-toc%255D&searchHistoryKey=#jp072540pAF2) *Changtao Xiao,*[*^†^*](http://pubs.acs.org/doi/abs/10.1021/jp072540p?prevSearch=%2528Gregory%2BScholes%2529%2BNOT%2B%255Batype%253A%2Bad%255D%2BNOT%2B%255Batype%253A%2Bacs-toc%255D&searchHistoryKey=#jp072540pAF2) *Yunfei Li,*[*^‡^*](http://pubs.acs.org/doi/abs/10.1021/jp072540p?prevSearch=%2528Gregory%2BScholes%2529%2BNOT%2B%255Batype%253A%2Bad%255D%2BNOT%2B%255Batype%253A%2Bacs-toc%255D&searchHistoryKey=#jp072540pAF3) *Xin Zhang,*[*^†^*](http://pubs.acs.org/doi/abs/10.1021/jp072540p?prevSearch=%2528Gregory%2BScholes%2529%2BNOT%2B%255Batype%253A%2Bad%255D%2BNOT%2B%255Batype%253A%2Bacs-toc%255D&searchHistoryKey=#jp072540pAF2) *Jialun Tang,*[*^†^*](http://pubs.acs.org/doi/abs/10.1021/jp072540p?prevSearch=%2528Gregory%2BScholes%2529%2BNOT%2B%255Batype%253A%2Bad%255D%2BNOT%2B%255Batype%253A%2Bacs-toc%255D&searchHistoryKey=#jp072540pAF2) *Shuai Chang,*[*^†^*](http://pubs.acs.org/doi/abs/10.1021/jp072540p?prevSearch=%2528Gregory%2BScholes%2529%2BNOT%2B%255Batype%253A%2Bad%255D%2BNOT%2B%255Batype%253A%2Bacs-toc%255D&searchHistoryKey=#jp072540pAF2)*^*^ Qibing Pei,*[*^‡^*](http://pubs.acs.org/doi/abs/10.1021/jp072540p?prevSearch=%2528Gregory%2BScholes%2529%2BNOT%2B%255Batype%253A%2Bad%255D%2BNOT%2B%255Batype%253A%2Bacs-toc%255D&searchHistoryKey=#jp072540pAF3) *and Haizheng Zhong*[*^†^*](http://pubs.acs.org/doi/abs/10.1021/jp072540p?prevSearch=%2528Gregory%2BScholes%2529%2BNOT%2B%255Batype%253A%2Bad%255D%2BNOT%2B%255Batype%253A%2Bacs-toc%255D&searchHistoryKey=#jp072540pAF2)

[†](http://pubs.acs.org/doi/abs/10.1021/jp072540p?prevSearch=%2528Gregory%2BScholes%2529%2BNOT%2B%255Batype%253A%2Bad%255D%2BNOT%2B%255Batype%253A%2Bacs-toc%255D&searchHistoryKey=#jp072540pAF2) Beijing Key Laboratory of Nanophotonics and Ultrafine Optoelectronic Systems, School of Materials Science & Engineering, Beijing Institute of Technology,5 Zhongguancun South Street, Haidian District, Beijing, 100081, China

‡ Department of Materials Sciences and Engineering, California NanoSystems Institute, Henry Samuli School of Engineering and Applied Science, University of California, Los Angeles, California, United States

* E-mail: [schang@bit.edu.cn](mailto:schang@bit.edu.cn)


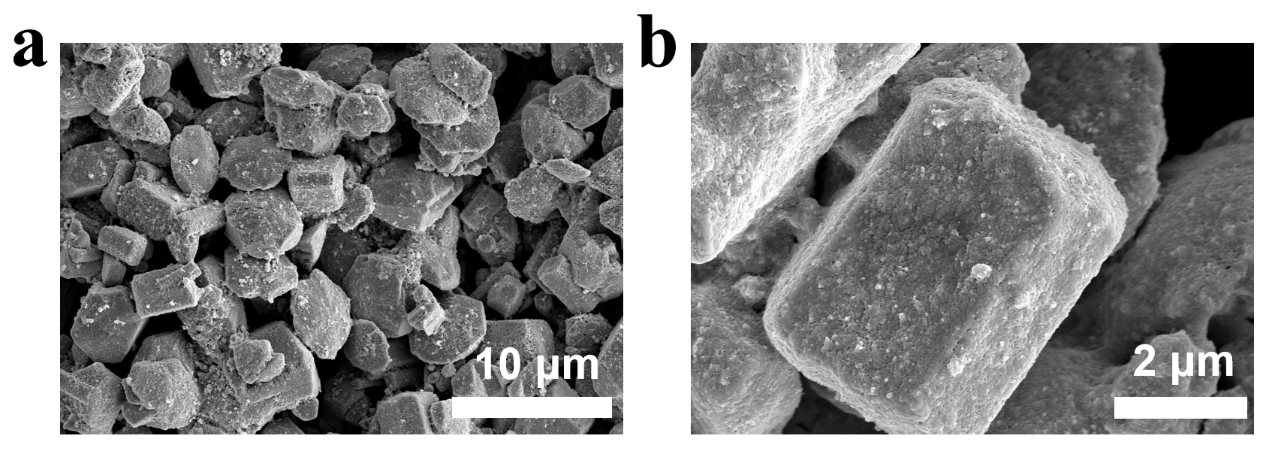


Figure S1. SEM images of precipitates obtained in conventional emulsion synthesis. (a) Scale bar of 10 μm; (b) Scale bar of 2 μm.


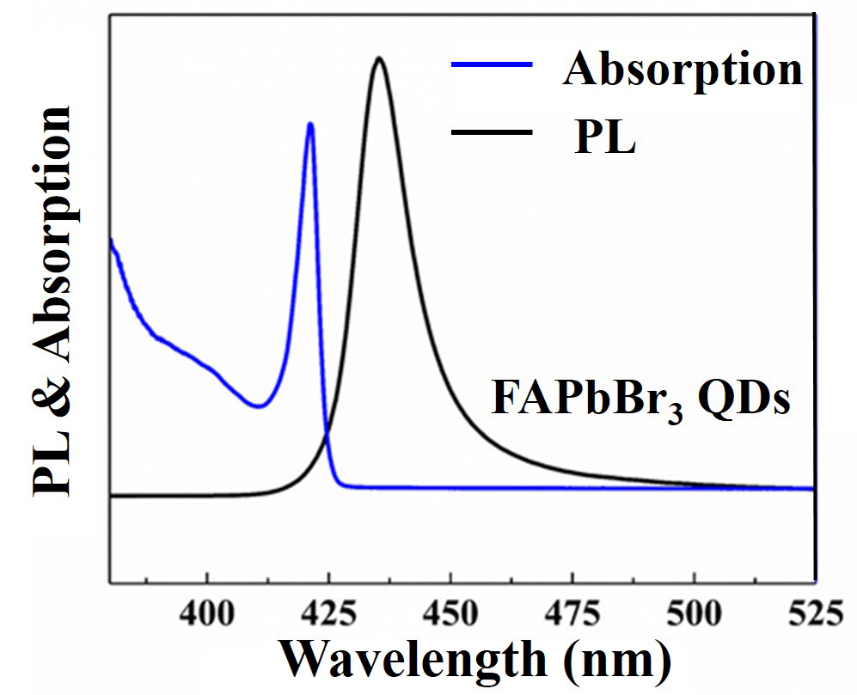


Figure S2. Absorption and PL spectra of the as-fabricated FAPbBr_3_ QDs.


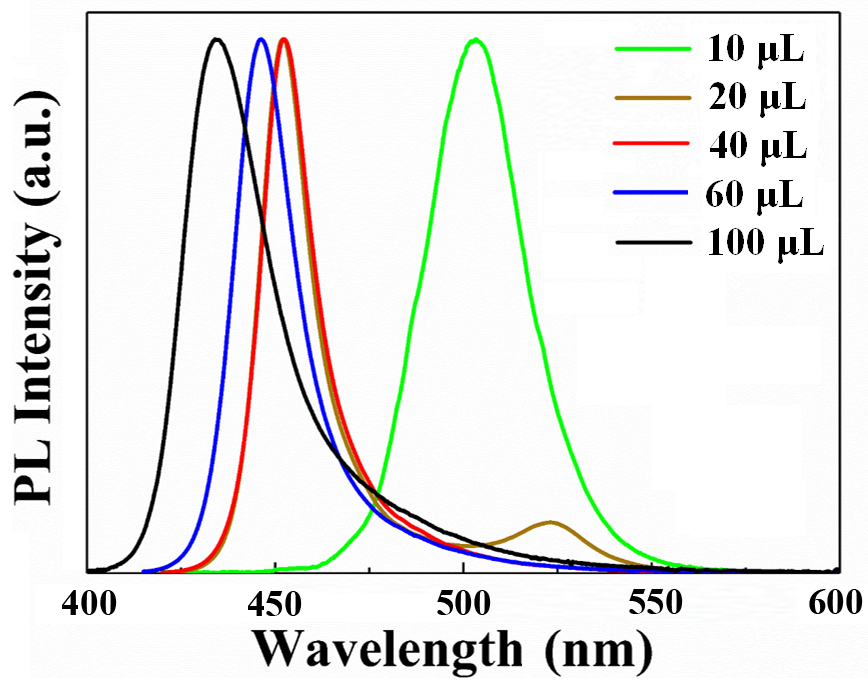


Figure S3. Wavelength tunable CH_3_NH_3_PbBr_3_ QDs by varying the amount of ligands.


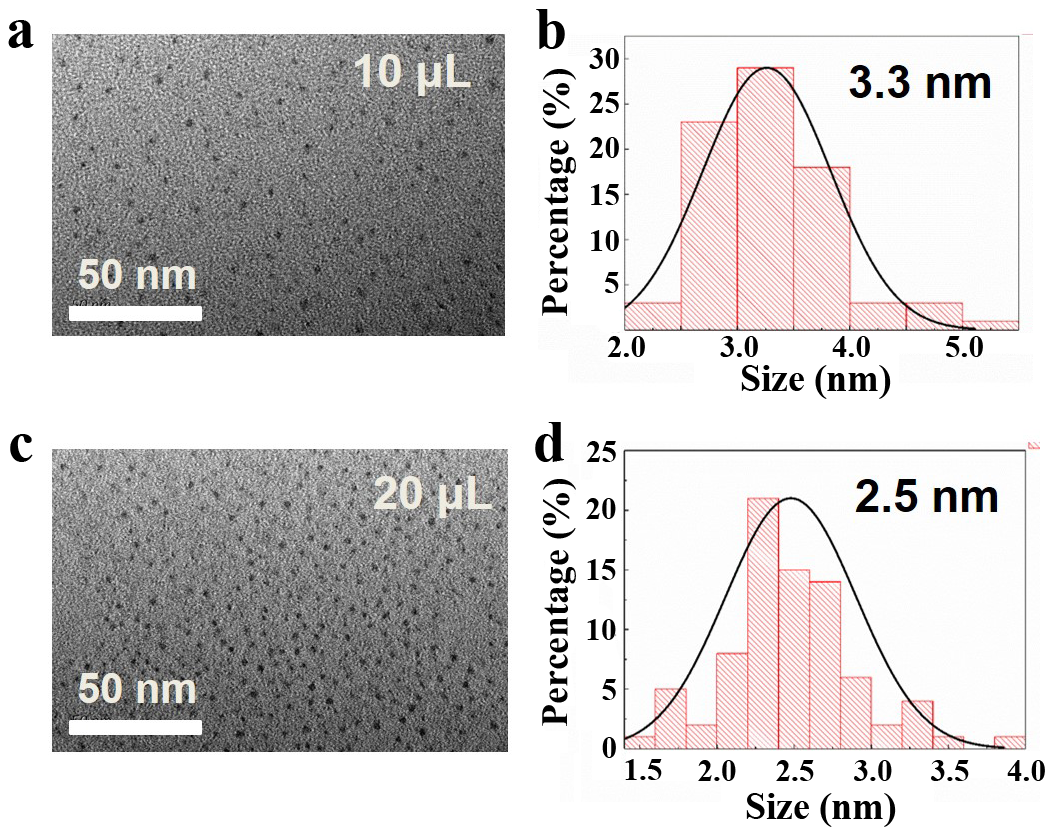


Figure S4. TEM images of CH_3_NH_3_PbBr_3_ QDs fabricated using varied amount of ligands and the corresponding statistical analysis of QD sizes. (a, b) 10 μL; (c, d) 20 μL.
